# Evaluate performance of the Abbott chemiluminescent microparticle immunoassay assay for detection of syphilis infection in Chinese blood donors

**DOI:** 10.1002/jcla.23033

**Published:** 2019-10-16

**Authors:** Ping Fu, Sushil Devare, Jian‐fang Liu, Xiao‐ting Lv, Peng Yin, Bing‐ting Wu, Ling Ke, Yu Liu, Hua Shan

**Affiliations:** ^1^ Institute of Blood Transfusion Chinese Academy of Medical Sciences & Peking Union Medical College Chengdu China; ^2^ Sichuan Blood Safety and Blood Substitute International Science and Technology Cooperation Base Chengdu China; ^3^ Abbott Diagnostics Lake Forest IL USA; ^4^ Abbott Diagnostics Shanghai R&D Center Shanghai China; ^5^ Transfusion Medicine Service Stanford University Medical Center Stanford CA USA

**Keywords:** blood donors, China, CMIA, syphilis

## Abstract

**Background and Objectives:**

To prevent *Treponema Pallidum* (TP) transmission from blood transfusion, enzyme‐linked immunosorbent assay (EIA) for anti‐TP has been widely used in routine blood donation screening in China for many years. The aim of this study was to evaluate the performance of the Abbott CMIA assay for detection of anti‐TP in Chinese blood donors.

**Materials and methods:**

A total of 2420 plasma samples, already routinely screened for anti‐TP by two different EIAs, from four blood Centers were tested for anti‐TP by Abbott CMIA. Subsequently, all samples with positive results by one or both EIAs and/or by Abbott CMIA were subjected to confirmatory testing (CT) using recombinant immunoblot assay (RIBA) or *Treponema Pallidum* particle agglutination assay (TPPA). TP infection was defined by a RIBA or TPPA positive.

**Results:**

Compared with two EIAs strategy, Abbott CMIA showed a relatively best sensitivity as 98.80% (95% CI: 97.44%‐100.16%) and a relatively best specificity as 99.58% (95% CI: 99.30%‐99.85%), yielding the best consistency (99.49%) between anti‐TP CT results with the highest *κ* value of .98.

**Conclusion:**

This is the first study to evaluate the performance of the Abbott CMIA assays for detection of syphilis in Chinese blood donors. Our results suggested that CMIA performed better than both EIAs, and implementation of CMIA replacing two different EIA reagents might help to further reduce the risk of transfusion‐transmitted TP infection, decrease unnecessary blood waste and loss of blood donors.

## 1 INTRODUCTION

Syphilis, a genital ulcerative disease caused by the bacterium *Treponema pallidum* (TP), is associated with significant complications if left untreated and can facilitate the transmission and acquisition of HIV infection. It is a curable sexually transmitted infection (STI).^1^ It can also be transmitted from mother to child in utero or during birth or, rarely, by transfusion of blood, blood components, or organs from donors with active syphilis.^2^, ^3^, ^4^, ^5^, ^6^ In 2008, approximately 11 million new cases of infection were reported worldwide,^7^ and 6.3 million new cases were estimated by World Health Organization (WHO) in 2016.^8^ Additionally, historical data demonstrate that untreated early syphilis in pregnant women, if acquired during the 4 years before delivery, can lead to infection of the fetus in up to 80% of cases and may result in stillbirth or death of the infant in up to 40% of cases.^9^ 988 000 pregnant women were estimated being infected with syphilis in 2016, resulting in over 350 000 adverse birth outcomes including 200 000 stillbirths and newborn deaths.^10^ In recent years, syphilis infection has become a serious problem in China. The total incidence of syphilis increased from 1.0 to 32.2 per 100 000 between 1995 and 2016 in China.^11^ A Nationwide Spatiotemporal Analysis of Syphilis in China indicated that the geographic distribution of syphilis incidence changed substantially during 2004‐2016, with a significant shift from coastal to inland provinces. In 2004, the highest incidence was reported in eastern China (17.3‐39.1/100 000), while there was a strong increase of syphilis in northwest China (15.3‐26.6/100 000) by 2010. Since 2010, the rising trend of syphilis in most Chinese provinces had reversed that incidence in eastern and southern central China has largely decreased (2.5‐25.1/100 000) while remaining high in northwest China (11.5‐25.6/100 000).^11^ Syphilis is a multistage disease with diverse and wide‐ranging manifestations, making laboratory testing a very important aspect of diagnosis. Currently, it is mainly relied on serological tests, including non‐*Treponema pallidum* serum tests (such as the Venereal Diseases Research Laboratory test (VDRL), the Rapid Plasma Reagin test (RPR), the Toluidine Red Unheated Serum Test (TRUST)), as well as the *Treponema pallidum* serum tests (such as *Treponema pallidum* Haemagglutination test [TPHA], Micro‐Haemagglutination Assay for TP [MHA‐TP], *Treponema pallidum* Passive Particle Agglutination test [TPPA], Fluorescent Treponemal Antibody absorption test [FTA‐abs test], Enzyme Immunoassay assay [EIA], and Chemiluminescence Immunoassay [CIA]).^12^ Due to its low detection limit, wide linear range, high precision, and shortened turnaround times, the chemiluminescent microparticle immunoassay (CMIA) technology is now widely applied to detect serum markers of Hepatitis B virus (HBV), Hepatitis C virus (HCV), TP, and Human immunodeficiency virus (HIV).^13^ CMIA has been used routinely in several developed countries in blood donor screening. Before 2015, the routine donor screening in China required parallel testing using two different EIA assays for each infectious disease marker tested. A reactive result in either one or both EIAs will disqualify the collected unit and defer the donor. In 2015, the new edition of Chinese National Standard Operational Protocol of Blood Centers approved the use of CMIA for anti‐TP detection in blood donor screening in China for the first time. We performed this study to evaluate and compare the performance of CMIA with currently used EIA in detecting anti‐TP in Chinese donors.

## 2 MATERIALS AND METHODS

This study was approved by the ethics committee of the Institute of Blood Transfusion (IBT), Chinese Academy of Medical Sciences.

### 2.1 Specimens

A total of 2420 donor samples were selected from The Recipient Epidemiology and Donor Evaluation Study‐III (REDS‐III) sample repository. All samples in REDS‐III repository had gone through routine screening for anti‐HCV, hepatitis B surface antigen (HBsAg), anti‐TP, and HIV‐1/2 Ab/Ag by two different licensed EIAs. Samples were selected randomly: These samples were from four different blood centers, and details of sample characteristics are shown in Table 1.

**Table 1:** Routine screening results of samples^a^

Routine anti‐syphilis screening results	Number of the samples (%)
Reactive by two anti‐TP EIAs	299 (12.36%)
Reactive by one anti‐TP EIA	126 (5.20%)
Gray zone^b^ by one anti‐TP EIA	2 (0.1%)
Gray zone^b^ by two anti‐TP EIAs	8 (0.33%)
Anti‐TP nonreactive with a screening reactive result in EIA for HBsAg, anti‐HCV or anti‐HIV	809 (33.43%)
Nonreactive in donor screening	1176 (48.60%)
Total	2420

a All samples were freshly frozen after collection before shipped to the Institute of Blood Transfusion (IBT) and restored at −20℃.

b Gray zone: samples tested with borderline results (0.5 < S/CO < 1) by EIA.

### 2.2 Anti‐TP screening by CMIA

The collected plasma samples were blinded to EIA results and assigned unique new sample identification numbers before tested with ARCHITECT Syphilis TP test (Abbott Diagnostics) on the automated chemiluminescent microparticle immunoassay analyzer ARCHITECT i2000 system (CMIA, Abbott Diagnostic). The ARCHITECT Syphilis TP assay is a two‐step immunoassay for qualitative detection of antibody to TP in human serum or plasma using CMIA technology with assay protocols, referred to as Chemiflex. All procedures were carried out according to manufacturer's instruction.^14^ Testing results were expressed as signal to cutoff (S/CO), and S/CO >1.0 is considered reactive. The sensitivity and the specificity of this assay are 100% and 99.76% respectively according to manufacturer's instruction.

### 2.3 Confirmatory testing

**Figure 1:**
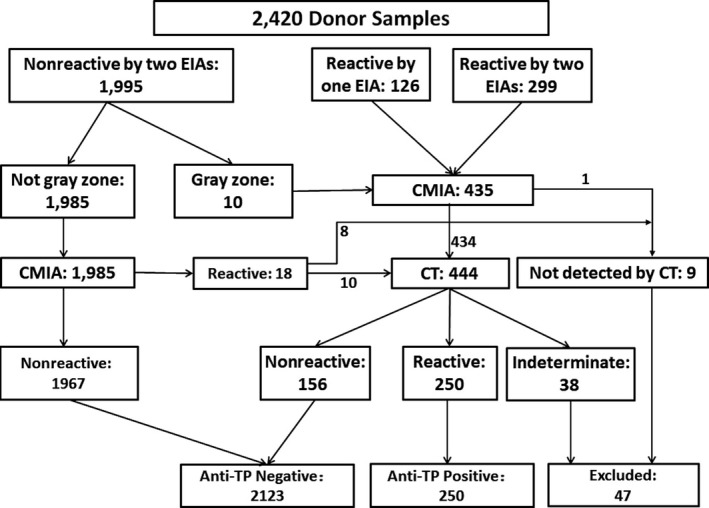
The algorithm of the screening and confirmatory testing. CMIA, Chemiluminescent microparticle immunoassay; CT, Confirmatory testing; EIA, Enzyme‐linked immunosorbent assay; TP, *Treponema Pallidum*

As recombinant immune blot assay (RIBA) and *Treponema pallidum* particle agglutination (TPPA) were considered as the gold standard reference methods, we used Mikrogen^®^ RIBA or FUJIREBIO^®^ TPPA to confirm anti‐TP screening results. Samples tested reactive by either one or two EIAs or CMIA were subjected to CT. The testing algorithm is shown in Figure 1.

### 2.4 Data analysis

Descriptive statistics were applied using the SPSS software (version 16.0) (SPSS Institute). The *κ* consistency test was used to compare consistency between screening results and CT results (*κ* < .40 indicated poor consistency; .40 ≤ κ < .75 indicated average consistency; and κ ≥ .75 indicated good consistency). Categorical variables were compared using the chi‐square test.

## 3 RESULTS

### 3.1 Results of the samples tested nonreactive for anti‐TP by two ELAs

A total of 1995 samples tested nonreactive for anti‐TP by two EIAs were tested by Abbot CMIA, including 10 anti‐TP gray zone (0.5 < S/CO < 1) and 809 screening reactive for other pathogens. Of these, 18 were reactive by Abbot CMIA. 10 of these 18 samples underwent CT: 4 (40%, 4/10) were confirmed reactive, 5 (50%, 5/10) were nonreactive, and 1 (10%, 1/10) were indeterminate. All 10 samples with EIA anti‐TP gray zone results were negative by Abbott CMIA. Nine of these 10 samples were subjected to CT and 1 (11.11%, 1/9) was indeterminate in CT. Data are detailed in Table 2.

**Table 2:** Results of the samples tested nonreactive for anti‐TP by two ELAs

Anti‐TP EIA results	Total	Abbott CMIA		CT results				
		+	−	Positive	Negative	IND	NT	Total
Negative	1176	3	1173	0	1	1	1	3
Gray zone^a^ by one EIA	2	0	2	0	0	1	1	2
Gray zone^a^ by two EIAs	8	0	8	0	8	0	0	8
anti‐TP nonreactive but HBsAg, anti‐HCV, or anti‐HIV reactive	809	15	794	4	4	0	7	15
Total	1995	18	1977	4	13	2	9	28

Abbreviations: −, Nonreactive; +, Reactive; IND, Indeterminate; NT, not tested.

a Gray zone: samples with border line results (0.5 < S/CO < 1).

### 3.2 Results of the samples tested reactive for anti‐TP by ELA

425 samples were screening reactive by anti‐TP EIA, including 299 reactive by both EIAs, 40 by EIA reagent 1, and 86 by EIA reagent 2. As shown in Table 3, out of the 425 samples, 61.88% (263/425) were reactive by Abbott CMIA and 38.12% (162/425) were nonreactive. Only 57.88% (246/425) were CT positive, 33.65% (143/425) were CT negative, and 8.47% (36/425) were CT indeterminate. Out of 126 samples, which were reactive by only one EIA reagent, only 7.94% (10/126) were confirmed anti‐TP positive.

**Table 3:** Results of the samples tested reactive for anti‐TP by EIA

CT	Total	Abbott CMIA		ELISA		
		+	−	R1+R2+	R1+R2−	R1−R2+
Positive	246	243	3	236	3	7
Negative	143	4	139	45	35	63
Indeterminate	36	16	20	18	2	16
Total	425	263	162	299	40	86

Abbreviations: −, Nonreactive; +, Reactive; R: EIA reagent.

### 3.3 Coincidence rate between CT results and EIAs or Abbott CMIA

In the EIA nonreactive group, 8 of the 18 CMIA reactive samples and 1 gray zone samples were not tested for CT due to insufficient sample volume. These samples were excluded for the coincidence rate analysis. 38 samples which were indeterminate in CT testing were also excluded, yielding 2373 samples for this analysis. 1967 samples which were nonreactive by both two EIAs and CMIA were not tested by CT and assumed to be anti‐TP negative, yielding a total of 250 anti‐TP confirmed positive cases and 2123 anti‐TP negative samples. As shown in Table 4, the data are displayed in four groups (CMIA, EIA reagent 1, EIA reagent 2, and two EIAs). Two EIA screening reagents correctly identified 246 samples as anti‐TP‐positive with a positive coincidence rate of 98.40% (95% CI: 96.83%‐99.97%) and 1980 samples as anti‐TP negative with a negative coincidence rate of 93.26% (92.20%‐94.33%). If 2373 samples were tested only by one screening reagent, Abbott CMIA EIA, reagent 1, and EIA reagent 2 correctly identified 247, 239, and 246 samples as anti‐TP positive; 2114, 2043, and 2015 samples as anti‐TP negative, respectively. Abbott CMIA showed the best positive coincidence rate of 98.80% (95% CI: 97.44%‐100.16%) and the best negative coincidence rate of 99.58% (95% CI: 99.30%‐99.85%), with a highest *κ* value of .98.

**Table 4:** Performance of different assays for anti‐TP screening

Different assays correctly identified	Confirmed positive n = 250 (%)	95% CI	Confirmed negative n = 2123 (%)	95% CI	Consistency	*κ*
Abbott CMIA	247 (98.80)	97.44‐100.16	2114 (99.58)	99.30‐99.85	99.49	0.97
EIA R1	239 (95.60)	93.04‐98.16	2043 (96.23)	95.42‐97.04	96.17	0.82
EIA R2	243 (97.20)	95.14‐99.26	2015 (94.91)	93.98‐95.85	95.15	0.78
Two EIAs	246 (98.40)	96.83‐99.97	1980 (93.26)	92.20‐94.33	93.81	0.74

## 4 DISCUSSION

The current practice at Chinese blood centers for serological screening of TP infection is by using two different EIAs in parallel and no routine confirmatory testing. If either of the two EIAs tested reactive, the donor is permanently deferred and the donated unit discarded. CMIA has been very widely used for clinical diagnosis and shown to be at least as sensitive and specific as the traditional techniques such as EIA.^15^, ^16^ CMIA has also been used routinely in developed countries for blood donor screening.

Numerous studies have reported that the anti‐TP ARCHITECT i2000SR CMIA has good sensitivity and specificity. The clinical performance of anti‐TP ARCHITECT i2000SR CMIA was investigated in China by testing of 1225 clinical specimens and reported the sensitivity as 99.16% and specificity as 98.99%.^17^ A study of 5543 specimens from Italy reported that the sensitivity of CMIA for the detection of anti‐TP was 100% and the specificity was 99.86%.^18^ In this study, we evaluated the performance of anti‐TP CMIA in blood donor samples from four Chinese blood centers using RIBA or TPPA as the confirmatory test. As shown in Table 4, in our study the Abbott CMIA demonstrated a relatively best sensitivity of 98.80% (95% CI: 97.44%‐100.16%) and a relatively best specificity of 99.58% (95% CI: 99.30%‐99.85%) when compared to the currently used two anti‐TP EIA strategy.

The two EIAs strategy was the mandated strategy for donor screening in China for many years and is still currently used by most Chinese blood centers. A recent change in government regulation now allows the use of CMIA for donor screening. As showed in Table 4, two EIAs in parallel showed an equivalent positive coincidence rate of 98.40% (96.83%‐99.97%), but it has the poorest negative coincidence rate of 93.26% (92.20%‐94.33%), which can result in unnecessary loss of blood and blood donors. The *κ* value was the intrinsic efficacy index among the reagents which was used to assess the degree of agreement between two tests.^19^ If Abbott anti‐TP CMIA were to replace the two EIA practice, it would result in the best consistency (99.49%) with the highest *κ* value of .98.

Out of 1995 samples screened anti‐TP nonreactive by two EIAs, 325 were HBsAg reactive, 240 were anti‐HCV reactive, and 238 were anti‐HIV reactive, of which 3, 5, and 7 were detected anti‐TP reactive by Abbott CMIA, respectively. It is interesting to note that out of 3 HBsAg reactive, 5 anti‐HCV reactive, and 7 anti‐HIV reactive samples, 0% (0/3), 25% (1/4), and 75% (3/4) were confirmed anti‐TP positive by CT respectively (data not shown). These cases of HIV and syphilis co‐infection again remind us the importance of effective syphilis detection in donor screening.

There are several limitations: (a) TPPA is recognized the “gold standard” of syphilis antigen serology test by WHO, historically having the best sensitivity of the treponemal tests, but actually had been shown to be less sensitive than EIA and CMIA; (b) the 1967 samples that were negative did not undergo confirmatory testing and were assumed to be anti‐TP negative, Therefore, discordances between the algorithms may be underestimated.

We report the first study to evaluate the performance of the Abbott CMIA assays for detection of syphilis in Chinese blood donors. Our results suggested that CMIA performed better than the two EIA approaches. The implementation of CMIA in routine donor may help to further reduce the risk of transfusion‐transmitted TP infection and decrease unnecessary blood waste.

## CONFLICT OF INTEREST

The authors Sushil Devare, Jian‐fang Liu, Xiao‐ting Lv, and Peng Yin are employees of Abbott Diagnostics; all other authors have no conflicts of interest.

## FUNDING INFORMATION

This work was supported by Abbott Diagnostics and the CAMS Innovation Fund for Medical Sciences (CIFMS, No.2016‐I2M‐1‐018).

## DISCLAIMER

None.
